# Eosinophil peroxidase activates cells by HER2 receptor engagement and β1-integrin clustering with downstream MAPK cell signaling

**DOI:** 10.1016/j.clim.2016.08.009

**Published:** 2016-10

**Authors:** Kerrie Hennigan, Paul J. Conroy, Marie-Therese Walsh, Mohamed Amin, Richard O'Kennedy, Patmapriya Ramasamy, Gerald J. Gleich, Zeshan Siddiqui, Senan Glynn, Olive McCabe, Catherine Mooney, Brian J. Harvey, Richard W. Costello, Jean McBryan

**Affiliations:** a Department of Medicine Respiratory Research Division, Royal College of Surgeons in Ireland, Beaumont Hospital, Dublin 9, Ireland; b Biomedical Diagnostics Institute, Dublin City University, Dublin 9, Ireland; c Department of Dermatology, University of Utah, Salt Lake City, USA; d Graduate Entry Medical School, University of Limerick, Ireland; e Department of Molecular Medicine, Royal College of Surgeons in Ireland, Beaumont Hospital, Dublin 9, Ireland; f Department of Physiology and Medical Physics, Royal College of Surgeons in Ireland, Dublin 2, Ireland

**Keywords:** Eosinophil peroxidase, HER2, β1-integrin, MUC4

## Abstract

Eosinophils account for 1–3% of peripheral blood leukocytes and accumulate at sites of allergic inflammation, where they play a pathogenic role. Studies have shown that treatment with mepolizumab (an anti-IL-5 monoclonal antibody) is beneficial to patients with severe eosinophilic asthma, however, the mechanism of precisely how eosinophils mediate these pathogenic effects is uncertain. Eosinophils contain several cationic granule proteins, including Eosinophil Peroxidase (EPO). The main significance of this work is the discovery of EPO as a novel ligand for the HER2 receptor. Following HER2 activation, EPO induces activation of FAK and subsequent activation of β1-integrin, via inside-out signaling. This complex results in downstream activation of ERK1/2 and a sustained up regulation of both MUC4 and the HER2 receptor. These data identify a receptor for one of the eosinophil granule proteins and demonstrate a potential explanation of the proliferative effects of eosinophils.

## Highlights

• Eosinophil peroxidase (EPO) is confirmed as a ligand for the HER2 receptor.
• EPO activation of HER2 leads to activation of FAK, ERK and β1 integrin.
• EPO induces a sustained upregulation of MUC4 and HER2.
• Possible mechanism for the proliferative effects of eosinophils is uncovered.

## 1 Introduction

Eosinophils accumulate at local inflammatory sites in allergic conditions such as asthma and allergic rhinitis [1], [2], [3], where they interact with resident cells including epithelial and nerve cells [4], [5], [6], [7]. IL-5 is a key mediator of eosinophil proliferation and activation, and studies have shown that treatment with mepolizumab and other anti-IL-5 monoclonal antibodies reduces the number of blood and sputum eosinophils [8], reduces exacerbations and improves quality of life in patients with refractory eosinophilic asthma [9], [10]. Eosinophils are also implicated in the pathogenesis of disease states such as inflammatory bowel disease [11], rhinitis [12], [13], helminth infections [14] and certain epithelial cancers [15] and play a role in non-pathological proliferative conditions such as normal mammary gland development [16]. Hence, eosinophils are implicated in the pathogenesis of a wide variety of human conditions. A suggested link between these pathological and physiological conditions is that eosinophils may play a role in regeneration and repair, although the contribution of the individual granule proteins to these processes is undefined [17].

Eosinophils contain four cationic granule proteins MBP, EDN, ECP and EPO, all of which are toxic at high concentrations to epithelial, muscle and neural cells as well as certain micro-organisms and parasites. However, at non-cytotoxic concentrations, eosinophil cationic proteins have been implicated in cell and tissue remodeling [18]. Other than a neural muscarinic receptor, no receptor that may mediate cell signaling has been identified for any of the eosinophil granule proteins [19].

Previous work in our laboratory, carried out in the neuroblastoma cell line IMR32, showed that EPO induced phosphorylation of HER2 at the Y1248 autophosphorylation site, and this led to loss of the cyclin-dependent kinase p27^kip^ from the nucleus and upregulation of the cell proliferation marker Ki67 [20]. These data suggest that EPO may be a ligand for HER2. The HER family consists of heavily glycosylated, single-chain modular cell surface glycoproteins [21], [22]. There are four structurally related family members which interact with each other; HER1 (EGFR/ErbB1), HER2 (neu/ErbB2), HER3 (ErbB3) and HER4. HER2 overexpression is notably associated with several human cancers, particularly breast and ovarian [23], [24], [25]. There is no known ligand for HER2, which acts as a co-receptor, forming heterodimers with other EGFR family members [26], whereas HER1, HER3 and HER4 are associated with one or more specific ligands [21]. MUC4, however, functions as a tethered endogenous HER2 ligand and influences HER2 activation [27], [28], [29]. In this study we examined the effect of EPO on human bronchial airway epithelial cells. We hypothesized, that EPO interacts with and activates the HER2 receptor with engagement and activation of the β1-integrin and then to downstream signaling events important for cell function, including inducing expression of the HER2 and MUC4 complex.

## 2 Materials and methods

### 2.1 Cell culture

Normal human bronchial epithelial 16HBE14o cells were maintained in MEM plus 10% FCS and 100 U/ml penicillin/streptomycin (GIBCO Invitrogen) and plated in the same medium but without FCS for experimentation. In some experiments cells were pre-treated with inhibitors, namely: AG825 (Calbiochem/Merck Biosciences) (10 μM, 2 h, inhibitor of HER2 activation), anti-CD29 (BD Biosciences) (1 μg/ml, 2 h β1 integrin inhibitor), PF573228 (TOCRIS Bioscience) (3 μM, 1 h, FAK inhibitor), or PNGase F (2 U/ml, 3 h, enzyme that catalyzes the complete removal of N-linked oligosaccharide chains from glycoproteins. Some cells were treated with EPO (4 μg/ml) or MBP(4 μg/ml) for various times.

### 2.2 Isolation of EPO and MBP

Eosinophil granule proteins were isolated from eosinophils of patients with marked eosinophilia as previously described [30], [31]. Briefly, sequential acid extractions were performed using 0.01 M HCl with extracts 1–3 containing > 80% of EPO. Extracts were then fractionated (1 ml fractions) on a Sephadex G-50 column. Absorbance at 280 nm revealed distinct peaks for EPO (fractions 36–50) and MBP (fractions 74–87). Fractions were further profiled by SDS-PAGE with Coomassie staining to confirm the homogeneity of each preparation.

### 2.3 Surface Plasmon resonance (SPR)

Surface Plasmon resonance (SPR) analysis was performed using a Biacore® 3000 and data analysis was carried out using BIAevaluation 4.1 software™. Analysis was carried out with research grade CM5 sensor chips using filtered degassed HBS-EP^+^ pH 7.4 (0.01 M HEPES, 0.15 M NaCl, 3 mM EDTA and 0.005% Tween-20®) as a running and sample buffer. All Biacore related consumables were purchased from GE Healthcare, Sweden. *Chip Preparation:* A research CM5 chip (GE Healthcare, UK) was prepared by immobilization of 7.5 μg/ml EPO in 10 mM sodium acetate, pH 4.2 using EDC-NHS coupling chemistry and 1 M ethylenediamine to cap unreacted sites [32] resulting in a chip preparation of 10,000RU of EPO. *Buffer screening:* Initially, the optimal binding buffers were examined to examine the interaction of recombinant HER2 and EPO. rHER2 (e-bioscience, UK) was reconstituted in molecular grade water (1 mg/ml) and was then prepared in a number of buffer compositions of varying NaCl, without EDTA or Tween-20, and also in standard HBS-EP^+^. To assess binding, the samples were injected for 3 min at 30 μl/min, dissociation monitored for 5 min and regenerated with a 30 s pulse of 5 mM NaOH. The data was collected and evaluated using the BIAevaluation 4.1 software package. *Binding Analysis:* HER2 (e-bioscience, UK) was reconstituted in 1 × HBS-EP^+^ (1 mg/ml) and was injected randomly at a number of concentrations (37.50, 18.75, 9.38, 4.99 and 0 nM), over the immobilized EPO for 3 min (flow rate 30 μl/min) and dissociation was monitored for 10 min. Regeneration of the surface was carried out as previously described. The data from the reference flow cell was subtracted to remove any systematic artefacts and each antigen response was then double-referenced by subtracting the buffer response (zero analyte concentration).

### 2.4 mRNA analysis

Total RNA was isolated from the cells with TRI reagent™ (Sigma) and reverse transcribed using QuantiTect Reverse Transcription cDNA synthesis kit (Qiagen, Hilden, Germany), according to the manufacturers' instructions. Quantitative RT-PCR analysis was carried out on the LightCyclerTM 1.0 (Roche) using QuantiTect SYBR Green PCR kit (Qiagen), according to the manufacturers' instructions as previously described [33]. Primers were specific to HER2, MUC4 or β-actin as normalizing gene (Table 1; Eurofins). Expression levels for each gene were quantified against serial dilutions of purified PCR product.

### 2.5 Protein preparation

Protein lysates were extracted in Relax buffer (100 mM KCl, 3 mM NaCl, 3.5 mM MgCl_2_, 10 mM HEPES, pH 7.4) containing protease and phosphatase inhibitor cocktails (Roche Diagnostics, Mannheim, Germany) and 1 × Triton. Lysates were disrupted by motorized pestle before nuclei were removed by centrifugation (1000*g* for 10 min) [34]. Protein concentration was established by the Bradford method [35].

### 2.6 SiRNA transfection

16HBE14o cells were serum-starved overnight prior to transfection with silencing RNA (30 nM final concentration) against HER2, FAK, GAPDH or a non-specific negative control silencing RNA (siRNA) (Applied Biosystems) in serum-free conditions, using Transfast (Promega) according to the manufacturers' instructions. Controls lacking siRNA were also set up. Down-regulation of the target genes was confirmed by Western blotting.

### 2.7 Western blotting

Protein (20, 30 or 65 μg, see individual figure legends) was subjected to SDS-PAGE electrophoresis and transferred overnight in Transfer buffer (150 mM Glycine, 20 mM Tris, 10%SDS, 2% Methanol) to nitrocellulose for Western blotting. Membranes were incubated in blocking buffer, (1 ×-Tris-buffered saline containing 5% skimmed milk powder (Fluka) and 0.1% (v/v) Tween-20), for 1 h at room temperature then blocking buffer containing 1:200 dilution of primary antibody FAK, pFAK or pERK, or 1:500 dilution of anti-active β1-integrin primary antibody (Millipore, CA) or ERK2 (Santa Cruz; Santa Cruz, CA, USA) or 1 ×-Tris-buffered saline containing 5% bovine serum albumin (Sigma) and 0.1% (v/v) Tween-20 containing 1:1000 dilution phospho-Y1248-HER2 or HER2 primary antibody (Cell Signaling Technology; Danvers, MA, USA), overnight at 4 °C with gentle rotation. After 6 times 5 min washes (Tris-buffered saline containing 0.1% (v/v) Tween-20), membranes were incubated for 2 h at room temperature in blocking buffer containing appropriate HRP-conjugated secondary antibody (Santa Cruz; 1:2000 dilution). After 6 times 5 min washes, membranes were exposed to Luminol Chemiluminescent reagent (Santa Cruz) for 1 min at room temperature followed by X-OMAT light sensitive film to obtain an image.

### 2.8 Immunofluorescence

16HBE14o cells were cultured on sterile coverslips, treated and fixed with 3.7% paraformaldehyde for 10 min at room temperature. Cells were incubated in blocking buffer (5% normal goat serum in 1 ×-PBS, 0.5% v/v Triton) for 1 h at room temperature. Cells were washed 3 times for 5 min in PBS between blocking, primary and secondary antibody incubations. Primary and secondary antibodies were diluted in blocking buffer (without Triton); anti-β1-integrin (JB1B) (Santa Cruz Biotech) 1:80 or mouse IgG (goat anti-mouse IgG – Texas Red) (Santa Cruz Biotech) 1:200. Phalloidin (Alexa 488) (Invitrogen) 1:1000 and Mouse IgG (Sigma) 1:80. Both primary and secondary antibody incubations were for 1 h at room temperature. Nuclear staining was carried out during mounting with VECTASHIELD Hard Set Mounting medium with DAPI (Vector Laboratories, Inc.). Images were obtained using an LSM710 Confocal Microscope (Zeiss Inc. UK).

### 2.9 Bioinformatic analysis

The EPO protein sequence (accession number P11678) was divided using a 10 residue sliding window into 706 peptides which were submitted to the PepSite webserver [36], with the PDB structure for HER2, 1N8Z [37], chain C. Five peptides were predicted to bind to HER2 (p < 0.05), all containing the peptide KLQPQR. The six residue peptide KLQPQR was predicted to interact with 1N8Z residues TYR C 252 and PHE C 257 (p < 0.05).

### 2.10 Statistical analysis

Values are expressed as mean ± SEM. Data was evaluated by two-tail Student's *t*-test or by ANOVA using the Graphpad Instat program. A *p* value of 0.05 or less was taken as significant.

## 3 Results

### 3.1 EPO is a ligand for HER2

**Fig. 1:**
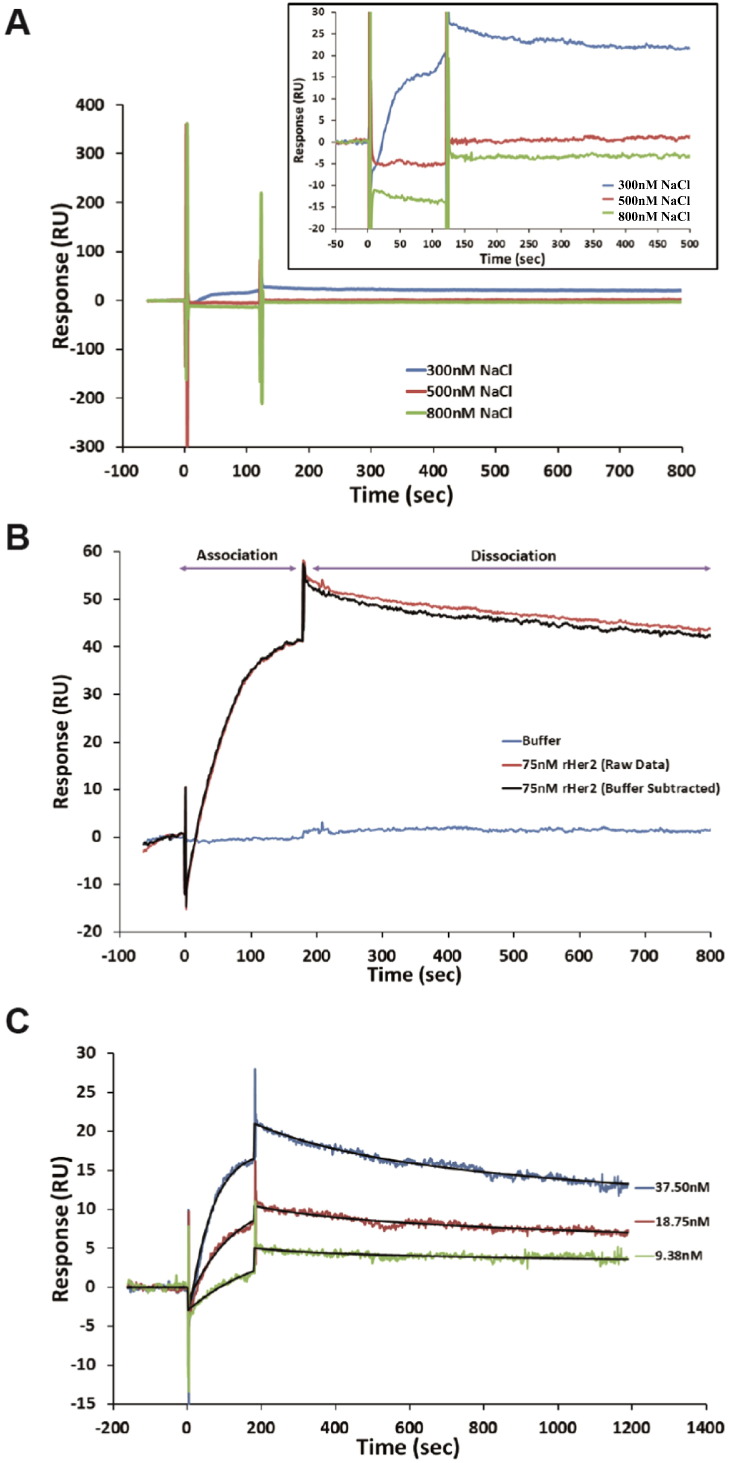
*Analysis of the interaction between EPO and rHER2 on Biacore* The analysis was carried out on immobilized EPO. (A) Double reference subtracted binding of 75 nM rHER2 to EPO was performed over a range of salt concentrations (300–800 mM). The binding of rHER2 was diminished between 300 and 500 mM NaCl suggesting the importance of charge for the interaction. The overlay box shows an expansion of each sample. In (B) the binding profile for rHER2 in HBS-EP^+^ is shown. The red trace is the 75 nm rHER2 and the blue trace 0 nM (buffer only) raw data (online reference cell subtracted). The black trace is the double reference subtracted data (75 nM rHER2 minus 0 nM response). (C) Kinetic analysis of three HER2 concentrations (double reference subtracted data shown) is fitted with a bivalent analyte model (black line) with local Rmax fitting. The kinetic evaluation suggested *k*_a1_ = 1.29 × 10^5^ M^− 1^ s^− 1^, *k*_d1_ = 2.03 × 10^− 3^ s^− 1^.

A recombinant form of the extracellular domain of HER2 (amino acids 23–652) was employed for SPR studies (rHER2, e-bioscience UK) to ascertain if EPO is a ligand for HER2. Due to its extremely positive charge, EPO (pI = 10.8) could not be orientated as the analyte and so was immobilized on the chip surface. The buffer composition, to minimize non-specific interactions, was examined and demonstrated that the interaction was influenced by NaCl concentrations above 300 mM (Fig. 1A) suggesting that the charge component of the proteins was a critical part of the binding process (in HBS pH 7.4). Varying the buffer pH reduced non-specific interaction between rHER2 and the reference flow cell (at rHER2 theoretical pI of 6.2) however, the binding curves took on a biphasic appearance. Reliable data was obtainable in HBS-EP^+^ (Fig. 1B) which minimized non-specific rHER2 interactions. Despite immobilizing various densities of EPO, the surface activity was relatively low which prevented rigorous analysis (densities at 4000, 8000 and 12,000 RU were examined). Binding analysis demonstrated a concentration dependent interaction between the rHER2 and EPO (Fig. 1C). As the extracellular domain of HER2 is known to be sufficient for ligand-independent homodimerization [38], this was expected to play a role in the interaction and consequently the data was fitted with the bivalent model for kinetic characterization. The derived binding constants suggested affinity *k*_a1_ = 1.29 × 10^5^ M^− 1^ s^− 1^, *k*_d1_ = 2.03 × 10^− 3^ s^− 1^ and *k*_a2_ = 6.15 × 10^− 4^ RU^− 1^, *k*_d2_ = 5.15 × 10^− 4^ s^− 1^.

**Fig. 2:**
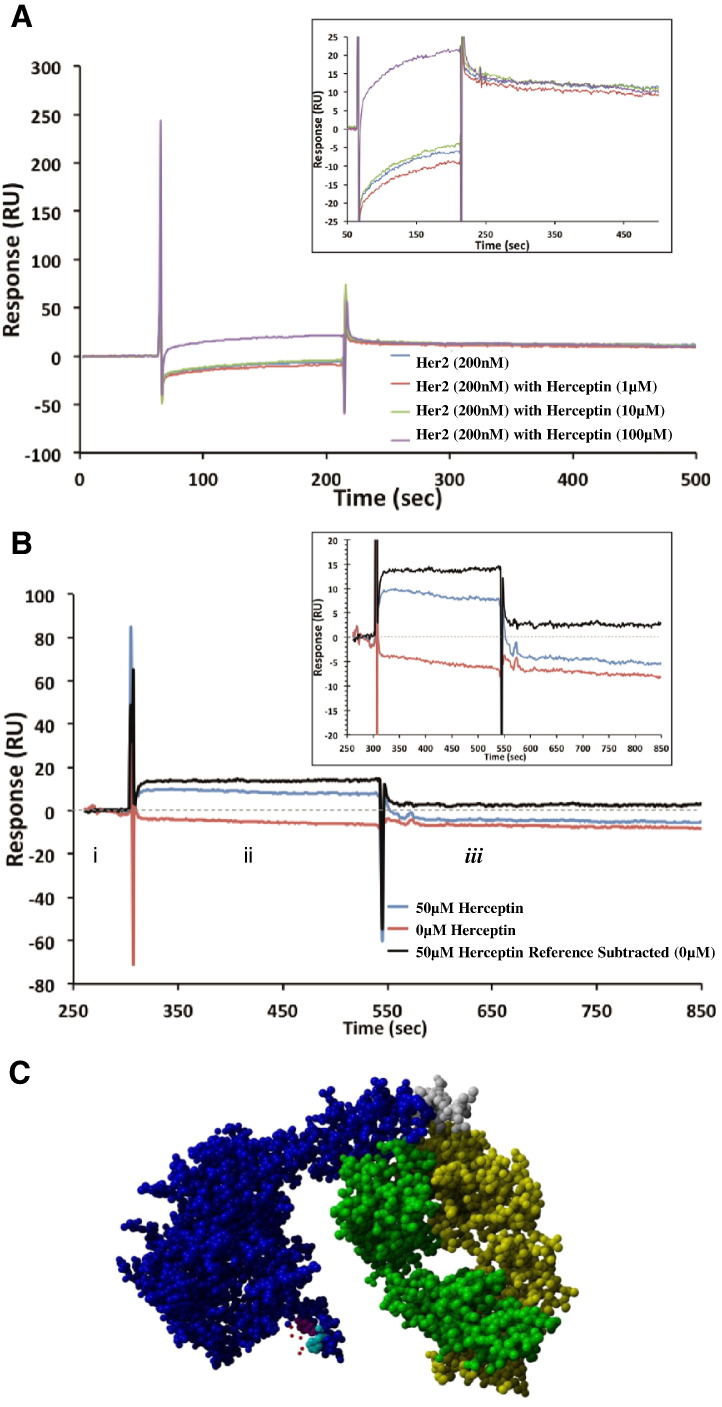
*Assessment of interaction of EPO-rHER2 and Herceptin* (A) Competitive binding profiles for EPO to rHER2 with incubation for 1 h with an excess of Herceptin. No diminishment of binding for EPO was observed in comparison to rHER2 with zero Herceptin. The overlay box shows an expansion of each sample. (B) Sequential injection of rHER2 across EPO (i) followed by Herceptin (ii) and monitoring of dissociation for 5 min (iii). The red trace is the dissociation of rHER2 from EPO (with no Herceptin) and the blue trace is the data collected after injection of Herceptin (50 μM). The black trace is the double referenced data (removing the dissociation of rHER2 from EPO). The overlay box is an expansion showing that Herceptin bound to rHER2 despite the receptor interacting with EPO. The results suggest that the binding site for EPO does not overlap with the known binding site for Herceptin (amino acid region 557–603 [37]). (C) 3D crystal structure of HER2-Herceptin complex with predicted binding site for EPO indicated. Blue and grey: extracellular domain of HER2 with grey indicating the amino acids closest to the transmembrane domain. Green and yellow: heavy and light chains of Herceptin antibody. Red: Amino acids of EPO which are predicted to interact with HER2. Magenta: HER2 amino acid 252. Cyan: HER2 amino acid 257.

To further examine the nature of the rHER2-EPO interaction, the monoclonal antibody to HER2, Herceptin, was used to shed light on the region of rHER2 involved in EPO binding. In competitive binding assays, a fixed rHER2 concentration incubated with excess of Herceptin (5, 50 and 500-fold) did not disrupt the interaction of EPO and HER2 and suggested that Herceptin and EPO do not share a common binding region (Fig. 2A). This was further examined by allowing rHER2 to bind EPO and subsequently, injecting Herceptin which showed small levels of binding, which could be attributed to lower rHER2 capture levels, and further implies non-overlapping binding sites (Fig. 2B).

Bioinformatic analysis with PepSite was used to predict potential polypeptide interactions between EPO and HER2. Only one specific region of the EPO amino acid sequence (KLQPQR, amino acids 103 to 108) reported a significant binding prediction for the extracellular domain of HER2 (p = 0.01726). The predicted interacting residues on the HER2 receptor structure are TYR 252 and PHE 257. As shown in the binding model in Fig. 2C, this predicted binding location for EPO is distinct from the known Herceptin binding location on HER2, consistent with experimental results.

### 3.2 Enzymatic removal of N-linked oligosaccharide chains prevents EPO-induced activation of HER2

**Fig. 3:**
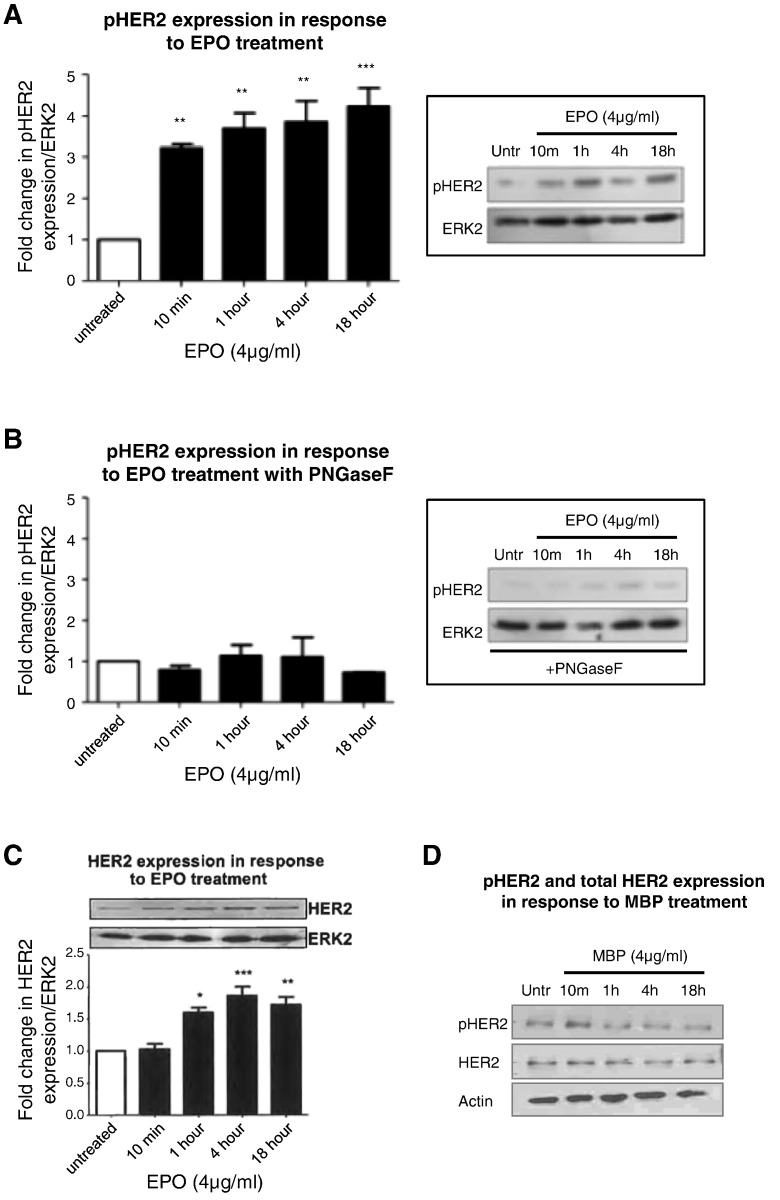
*EPO induces HER2 phosphorylation in an N-linked glycosylation-dependent mechanism* (A) 16HBE14o cells were exposed to EPO (4 μg/ml) for varying times and fold change in pHER2 (at its autophosphorylation site Y1248) was assessed by Western blotting. (B) 16HBE14o cells were first pretreated with an enzyme that catalyzes the complete removal of N-linked oligosaccharide chains from glycoproteins (PNGase F, 2 U/ml, 1 h) and then exposed to EPO (4 μg/ml) for varying times. Fold change in pHER2 was assessed. (C) 16HBE14o cells were exposed to EPO (4 μg/ml) for varying times and fold change in HER2 was assessed by Western blotting. The graphs show the fold change in pHER2 (A,B) or HER2 (C) expression levels in response to EPO treatment compared to untreated cells in the absence (A,C) or presence (B) of PNGase F at the indicated times. (n = 3, mean ± sem; *p < 0.05, **p < 0.01, ***p < 0.001). (D) 16HBE14o cells were exposed to MBP (4 μg/ml) for varying times and expression of pHER2 and HER2 was assessed by Western blotting. Images are representative of n = 3 experiments.

In functional studies, EPO (4 μg/ml) induced a rapid and sustained phosphorylation of the HER2 receptor (Fig. 3A). Prior studies have shown that N-linked glycosylation (NLG) is a critical step in the post translational modification of RTKs including HER2 [39], [40]. Hence, 16HBE14o cells were pretreated with an enzyme that catalyzes the complete removal of N-linked oligosaccharide chains from glycoproteins (PNGase F, 2 U/ml, 3 h) and then exposed to EPO for varying times. These studies showed that there was no longer an induction in the phosphorylation of HER2 (Fig. 3B). EPO induced an upregulation of total HER2 receptor expression at a protein level (Fig. 3C). The increased phosphorylation of HER2 by EPO is not due simply to the increase in overall HER2 levels as the EPO-induced increase in phosphorylated HER2 occurs as early as 10 min (Fig. 3A), whereas a similar EPO-induced upregulation of the HER2 receptor, only occurs at 4 h (Fig. 3C). EPO had no effect on the transcriptional expression of HER1 or MUC1 at any timepoint (data not shown). MBP, another eosinophil cationic protein isolated by similar methodology to EPO, had no effect on levels of total or phosphorylated HER2, indicating the specificity of the HER2 response to the eosinophil granule protein EPO (Fig. 3D).

### 3.3 EPO induces a HER2-dependent activation of β1-integrin

**Fig. 4:**
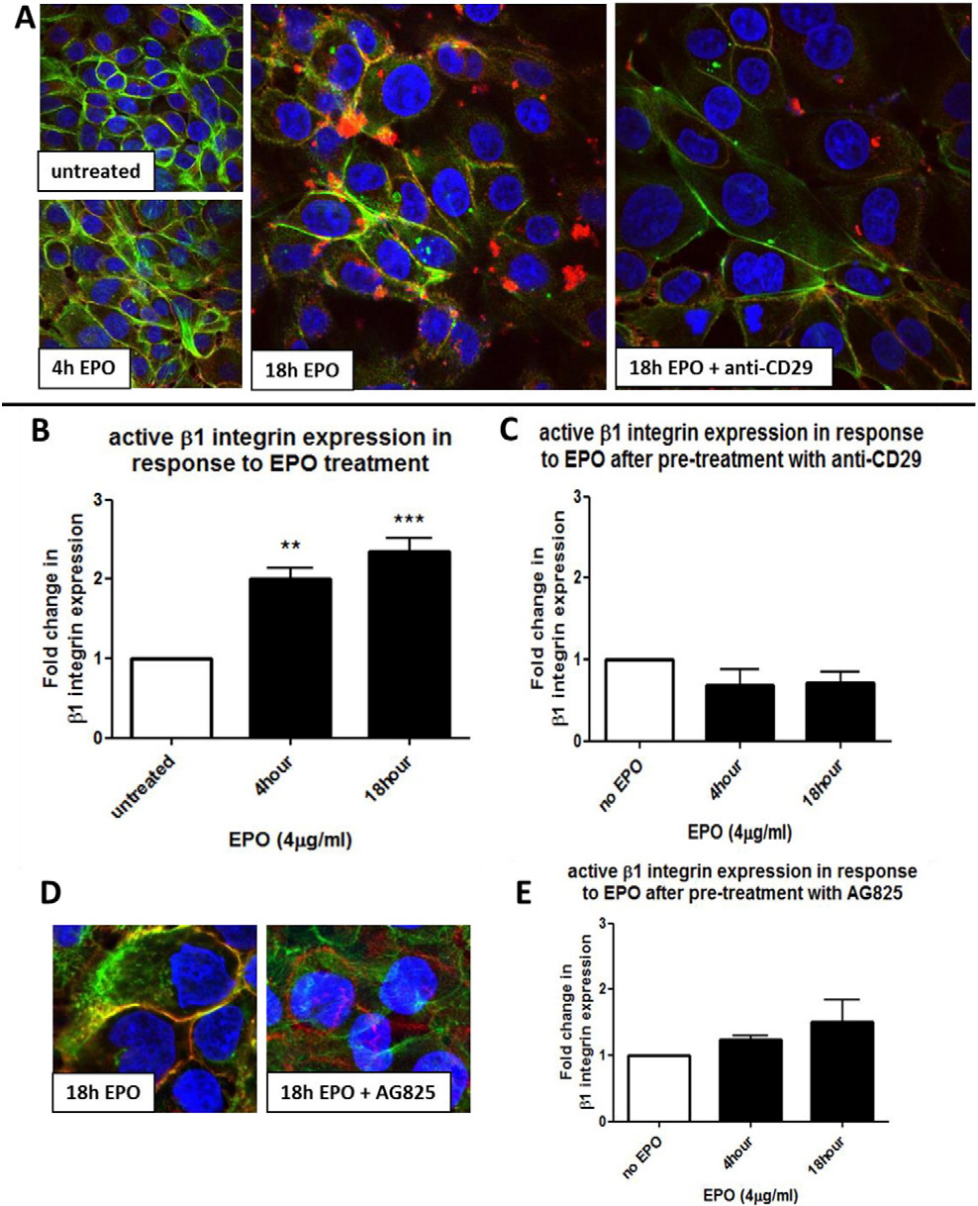
*EPO induces a pHER2-dependent activation of β1 integrin* (A, D) 16HBE14o cells were grown on coverslips and treated with EPO (4 μg/ml) for various times or left untreated (as indicated). Some wells were pre-treated with a specific anti-β1-integrin neutralizing antibody (anti-CD29, 1 μg/ml, 2 h) or pretreated with a HER2 tyrosine kinase inhibitor (AG825, 10 μM, 2 h), as indicated on images. β1-integrin (red) was stained with a mouse integrin-β1 antibody (JB1B, Santa Cruz) and a Texas Red goat anti-mouse IgG secondary antibody. β1-integrin activation is indicated by clustering and co-localization with F-actin fibers, stained with Phalloidin (green), resulting in the orange clusters visible at 18 h EPO treatment (A). In (B) cells were treated with EPO (4 μg/ml) for varying time points, cellular protein was subjected to Western blot analysis and probed for active β1-integrin and against ERK2 for normalization. In (C) cells were first pre-treated with anti-CD29 (1 μg/ml, 2 h) and then exposed to EPO (4 μg/ml) for varying time points. Cellular protein was subjected to Western blot analysis and probed for active β1-integrin and against ERK2 for normalization. In (D) confocal microscopy images show the comparison of cells treated with EPO for 18 h in the absence or presence of AG825 (as indicated). β1-integrin (red) activation is indicated by clustering and co-localization with F-actin fibers. In (E) 16HBE14o cells were first pre-treated with AG825 (10 μM, 2 h) and then exposed to EPO (4 μg/ml) for varying time points. The graphs show the fold change in active β1-integrin expression levels in response to EPO treatment compared to untreated cells in the absence (B) or presence (C, E) of inhibitors at the indicated times. (n = 3, mean ± sem; **p < 0.01, ***p < 0.001).

To determine if EPO-induced HER2 activation had further consequences for bronchial epithelial cells, we examined β1-integrin activation in response to EPO treatment. Confocal microscopy showed that, compared to untreated cells, EPO (4 μg/ml) induced the activation of β1-integrin (red staining) at 4 h and 18 h (Fig. 4A). These results were confirmed by Western Blotting using an antibody specific to the active form of β1-integrin (Fig. 4B). Pre-treatment with a neutralizing antibody to β1-integrin, (anti-CD29, 1 μg/ml, 2 h) (Fig. 4C) confirmed that the clusters observed in Fig. 4A were β1-integrin. EPO-induced integrin activation is mediated via HER2, since pre-treatment with AG825 (chemical inhibitor of the tyrosine kinase activity of HER2) prevented activation of β1-integrin observed by confocal fluorescence imaging and Western blot (Fig. 4D and E).

### 3.4 EPO-induced FAK and ERK activation

**Fig. 5:**
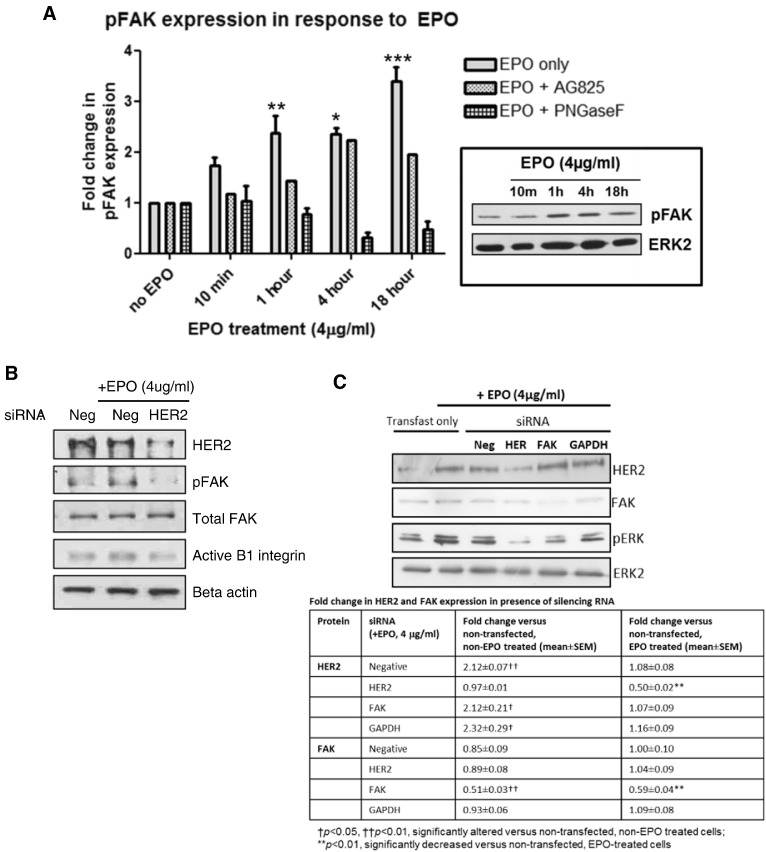
*EPO induces the activation of focal adhesion kinase (FAK) and HER2 which are required for activation of β1 integrin* (A) Western blots of 16HBE14o protein from cells treated with or without EPO (4 μg/ml) at the indicated times. Some were pre-treated with the inhibitor AG825 (10 μM, 2 h) or the endoglycosidase PNGase F (2 U/ml, 1 h) (see legend) and probed with a rabbit anti-human pFAK antibody. The graphs show the fold change in pFAK expression levels in response to EPO treatment compared to untreated cells in the absence or presence of inhibitors at the indicated times. (n = 3, mean ± sem; *p < 0.05, **p < 0.01, ***p < 0.001). The blots shown are representative of 3 similar experiments. Protein levels of total FAK remain unchanged in the presence of EPO and the various inhibitors studied (data not shown). (B) Western blots of 16HBE14o cells grown in the absence or presence of EPO (4 μg/ml, 18 h) as indicated. Cells were transfected with silencing RNA (siRNA) to a scrambled negative control (Neg) or HER2. (C) Western blots of 16HBE14o cells grown in the absence or presence of EPO (4 μg/ml, 18 h) as indicated. Cells were transfected or not with siRNA to a scrambled negative control (Neg), HER2, FAK or GAPDH. Table shows the fold change in HER2 and FAK protein expression following transfection.

The downstream signaling consequences of EPO-induced HER2 activation were then investigated. Western Blotting showed that EPO (4 μg/ml) induced the phosphorylation of FAK in a time-dependent manner from as early as 10 min (Fig. 5A). This EPO-induced FAK activation was inhibited by AG825 indicating its dependence on the initial activation of HER2. In further support of this, silencing RNA against HER2 reduced the levels of phosphorylated FAK and also activated β1-integrin under EPO-treated conditions (Fig. 5B). The EPO induced FAK phosphorylation was also reduced by PNGase F pre-treatment (Fig. 5A).

**Fig. 6:**
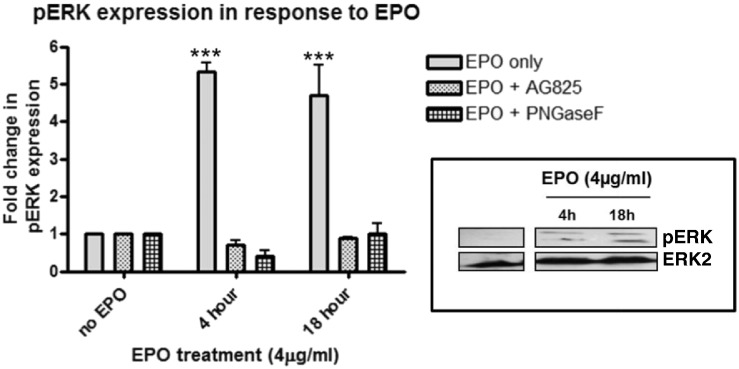
*EPO induces the activation of extracellular signal-regulated kinase (ERK) via N-linked glycosylation through the initial activation of HER2* Western blots of 16HBE14o protein from cells treated with or without EPO (4 μg/ml) at the indicated times. Some were pre-treated with the inhibitor AG825 (10 μM, 2 h) or the endoglycosidase PNGase F (2 U/ml, 1 h) (see legend) and probed with a rabbit anti-human pERK antibody. The graphs show the fold change in pERK expression levels in response to EPO treatment compared to untreated cells in the absence or presence of inhibitors at the indicated times. (n = 3, mean ± sem; *p < 0.05, **p < 0.01, ***p < 0.001). The blots shown are representative of 3 similar experiments. Protein levels of total ERK remain unchanged in the presence of EPO and the various inhibitors studied (data not shown).

Silencing RNA directly targeted against FAK did not impact EPO-induced HER2 expression (Fig. 5C). However, silencing RNA against either FAK or HER2 reduced phospho ERK expression under EPO treated conditions, indicating that ERK activation is a downstream target of this pathway (Fig. 5C). Indeed, EPO treatment (4 μg/ml) was shown to induce the phosphorylation of ERK1/2 in a time-dependent manner from as early as 10 min and this was inhibited by both AG825 and PNGase F pretreatment, further indicating its dependence on initial EPO-induced activation of HER2 (Fig. 6).

### 3.5 EPO regulates the transcriptional levels of HER2 and MUC4

**Fig. 7:**
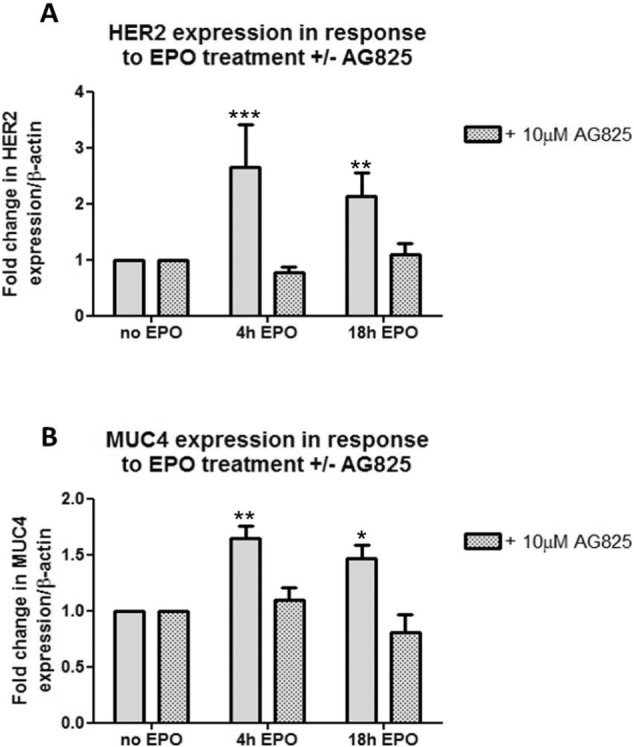
*EPO induces a pHER2-dependent increase in the transcriptional levels of HER2 and MUC4* 16HBE14o cells were treated with EPO (4 μg/ml) in the absence of or following pre-treatment with AG825 (10 μM, 2 h). Cellular material was harvested for RNA isolation and cDNA synthesis and Real-time PCR was performed. Graphs show the fold change in HER2 (A) or MUC4 (B) relative to β-actin expression in response to EPO (4 μg/ml) at the indicated time points compared with untreated cells (n = 3, mean ± sem; *p < 0.05, **p < 0.01, ***p < 0.001).

EPO (4 μg/ml) induces an increase in transcriptional expression of HER2. This was mediated via the initial activation of the HER2 receptor since EPO-induced HER2 upregulation was inhibited when cells were pre-treated with AG825 (10 μM, 2 h) (Fig. 7A). MUC4 is a tethered ligand for HER2 influencing its activation [27]. Real-time PCR showed that EPO induced an upregulation in the transcriptional expression of MUC4 (Fig. 7B). Furthermore, in the presence of AG825, the levels of MUC4 did not increase in response to EPO treatment (Fig. 7B), indicating that EPO-induced upregulation in MUC4 gene expression was dependent on the initial activation of the HER2 receptor.

## 4 Discussion

**Fig. 8:**
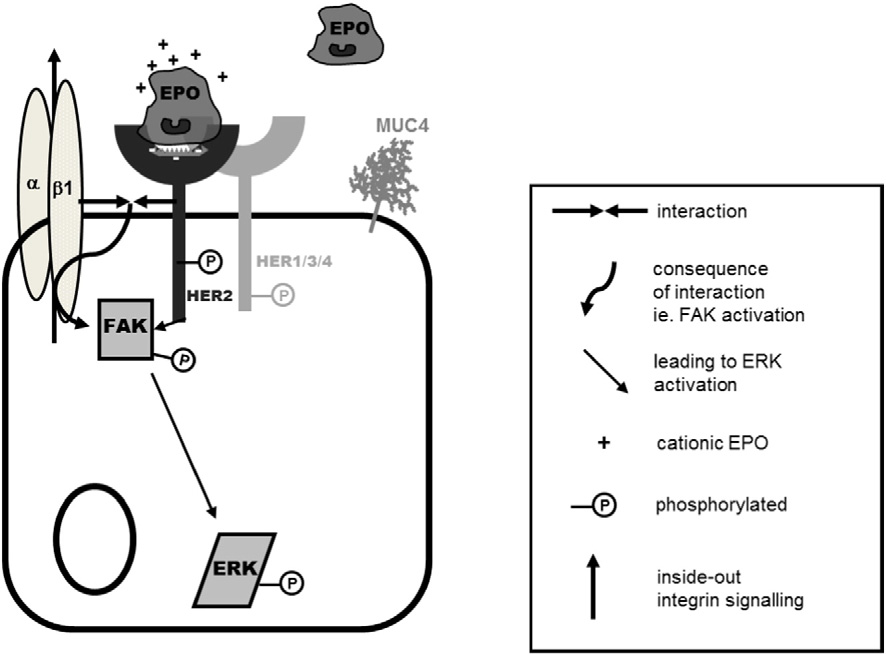
*Summary diagram* EPO binds to the HER2 receptor, resulting in its activation, which in turn activates FAK. FAK acts as a scaffold protein bringing together β1-integrin and the HER2 receptor with subsequent HER2-dependent, FAK-dependent β1-integrin activation. EPO also induces a pHER2-dependent, FAK-dependent activation of ERK 1/2.

In this study we have demonstrated that the eosinophil granule protein EPO is a ligand for the HER2 receptor. EPO engages HER2 via a site separate to that bound by the receptor inhibitor Herceptin and potentially via amino acids 252 and 257 of HER2. Ligand binding of EPO to HER2 results in phosphorylation of the HER2 receptor with subsequent phosphorylation of the scaffold protein, FAK and inside-out activation of β1-integrin. Together this complex of HER2, FAK and β1-integrin leads to activation of the MAP kinase ERK1/2 with a subsequent increase in HER2 receptor and MUC4 mRNA expression (Fig. 8). These data imply a mechanism for the recognized proliferative and remodeling effects of EPO [41].

This study was designed to investigate how eosinophil granule proteins interact with cells. To date a vast amount of data has been reported on how eosinophils accumulate at specific sites of inflammation [42] and there is important historic evidence of a non-specific charge-mediated toxicity by these proteins [43], [44]. In our prior work, we and others have shown that at even higher concentrations than studied here, of up to 10 microgram/ml (1–3 log order lower than those that are toxic) the granule proteins MBP1 and EPO prevent cell necrosis and apoptosis [45], [46] via different mechanisms, indicating that the concentrations we used in these experiments are not toxic to these cells. Indeed, both granule proteins induce quite separate intracellular signaling pathways with different cellular consequences [20], [47]. However, there has been little insight into the specific cellular receptors for these granule proteins, and this investigation is the focus of this work.

We investigated the interaction of EPO and HER2 using SPR. This, in vitro, technique facilitated investigation of the binding and the relative strength of that binding to the HER2 receptor and we used inhibitors which have known binding sites, to investigate the possible sites of interaction of EPO with HER2. EPO interacted with the HER2 receptor, and the strength of the interaction was calculated using a bivalent analyte model. It is worth noting that the high charge of the interaction pair was a barrier to examination of the interaction in a 1:1 fashion (with EPO as the analyte). To further investigate the nature of the interaction, Herceptin's ability to inhibit the interaction was examined. Herceptin is known to interact with three regions of HER2, which are towards the C-terminus (amino acids 557–561, 570–573 and 593–603) [37]. From the analysis, we concluded that the interaction of EPO was unaffected by Herceptin binding suggesting that EPO binds within a different region of HER2. This finding is further supported by mathematical modelling which predicts the most likely interaction between EPO peptide sequence and the 3D structure of HER2 is at amino acids 252 and 257 of HER2.

Incubation with EPO led to activation of the HER2 receptor, at its autophosphorylation site Y1248, a critical site of tyrosine kinase activity [48]. EPO also increased the levels of expression of the HER2 receptor in a manner that was dependent on the initial phosphorylation of the receptor, suggesting that this may be a pro-proliferative effect of EPO. The increased phosphorylation of HER2 by EPO is not due simply to the increase in overall HER2 levels as the EPO-induced increase in phosphorylated HER2 occurs as early as 10 min, whereas a similar EPO-induced upregulation of the HER2 receptor only occurs at 4 h. Prior work has shown that N-linked glycosylation (NLG) is needed for proper membrane insertion, ligand binding and receptor functioning of HER2. All observed effects of EPO were inhibited by the endoglycosidase, PNGase F [49].

Integrins can facilitate growth factor-mediated activation of ERK [50] and FAK (whose pathway is activated by integrins and growth factor receptors) and is linked to signaling pathways that modify the cytoskeleton and activate mitogen-activated protein kinase (MAPK) cascades. Integrin clustering and association with the cytoskeleton appears to give rise to integrin-growth factor receptor complexes [50], [51]. The interactions and interdependence of growth factor receptors such as the HER family and integrins are underscored by observations such that β1-integrin can facilitate bypassing of the anti-proliferative effects of the HER2 neutralizing antibody, trastuzumab, in breast cancer cells [52]. In the current experiments EPO also activated β1-integrin, albeit indirectly, via pHER2-mediated activation of FAK. We found, through the use of silencing RNA experiments that when HER2 is silenced, the activation of β1-integrin is reduced. While we recognize the limitations of using one single time-point for this data and agree that it does not fully complete the argument, in the context of the rest of this work, it is supportive. The formation of this scaffold is important for activation of β1-integrin, for interaction with the HER2 receptor and potentially for activation of further downstream signaling molecules, for example ERK MAP Kinase signaling. The EPO-induced activation of both FAK and ERK was also dependent on NLG.

Further downstream, we have shown that EPO induces an increase in the transcriptional expression of the HER2 receptor as well as mucin gene MUC4. EPO-induced upregulation of MUC4 and HER2 is of particular interest. MUC4 is a high-molecular weight glycoprotein that has been implicated in cancer progression particularly due to its cell signaling and anti-adhesive properties. MUC4 also interacts with HER2 by stabilizing the HER2 receptor and through activation of certain HER2 domains. MUC4 and HER2 interactions are of importance in the progression of a variety of epithelial tumors including breast cancer and pancreatic cancers, where eosinophils, in particular EPO are also a feature [27].

The main significance of this work is the discovery of EPO as a novel ligand for the HER2 receptor. The activation of the HER2 receptor by EPO is interesting because both EPO and HER2 are upregulated in certain disease states. For example, HER2 is associated with particularly aggressive forms of breast cancer [53] and pancreatic cancer [54]. Because eosinophils are implicated in chronic pancreatitis [55], one can speculate that chronic eosinophil activation in tissues may contribute to the development of malignancy. EPO, by activating HER2 and subsequent ERK activation may contribute to cell and tissue repair and also to cell proliferation which may mediate effects on mucous production. Thus, this is a rationale to investigate inhibitors of these pathways, when eosinophil tissue presence is prolonged or in conditions where eosinophil concentrations are elevated. Such studies could potentially allow us to provide intervention treatment before EPO tips the balance from being helpful to becoming pathological.

## Figures and Tables

**Table 1:** Primers.

Primer	Sequence	Melting temperature
HER2 forward	5′-CAG CAG AGG ATG GAA CAC AG-3′	59.4
HER2 reverse	5′-ACT CCT GGA TAT TGG CAC TG-3′	57.3
MUC4 forward	5′-CTG TGT CTC TGC CTC CTT CC-3′	61.4
MUC4 reverse	5′-TTG TTG AGC CTG TTG AGG TG-3′	57.3
β-actin forward	5′-GGA CTT CGA GCA AGA GAT GG-3′	59.4
β-actin reverse	5′-AGG AAG GAA GGC TGG AAG AG-3′	59.4
